# Susceptibility of *Anopheles stephensi* to *Plasmodium gallinaceum*: A Trait of the Mosquito, the Parasite, and the Environment

**DOI:** 10.1371/journal.pone.0020156

**Published:** 2011-06-09

**Authors:** Jen C. C. Hume, Howard Hamilton, Kevin L. Lee, Tovi Lehmann

**Affiliations:** Laboratory of Malaria and Vector Research, National Institute of Allergy and Infectious Diseases, National Institutes of Health, Rockville, Maryland, United States of America; Smithsonian Institution National Zoological Park, United States of America

## Abstract

**Background:**

Vector susceptibility to *Plasmodium* infection is treated primarily as a vector trait, although it is a composite trait expressing the joint occurrence of the parasite and the vector with genetic contributions of both. A comprehensive approach to assess the specific contribution of genetic and environmental variation on “vector susceptibility” is lacking. Here we developed and implemented a simple scheme to assess the specific contributions of the vector, the parasite, and the environment to “vector susceptibility.” To the best of our knowledge this is the first study that employs such an approach.

**Methodology/Principal Findings:**

We conducted selection experiments on the vector (while holding the parasite “constant”) and on the parasite (while holding the vector “constant”) to estimate the genetic contributions of the mosquito and the parasite to the susceptibility of *Anopheles stephensi* to *Plasmodium gallinaceum*. We separately estimated the realized heritability of (i) susceptibility to parasite infection by the mosquito vector and (ii) parasite compatibility (transmissibility) with the vector while controlling the other. The heritabilities of vector and the parasite were higher for the prevalence, i.e., fraction of infected mosquitoes, than the corresponding heritabilities of parasite load, i.e., the number of oocysts per mosquito.

**Conclusions:**

The vector's genetics (heritability) comprised 67% of “vector susceptibility” measured by the prevalence of mosquitoes infected with *P. gallinaceum* oocysts, whereas the specific contribution of parasite genetics (heritability) to this trait was only 5%. Our parasite source might possess minimal genetic diversity, which could explain its low heritability (and the high value of the vector). Notably, the environment contributed 28%. These estimates are relevant only to the particular system under study, but this experimental design could be useful for other parasite-host systems. The prospects and limitations of the genetic manipulation of vector populations to render the vector resistant to the parasite are better considered on the basis of this framework.

## Introduction

Vector-borne diseases such as malaria, filariasis, and dengue top the public health priority of countries around the world [1]. Highly mobile, abundant, and well-adapted for exploiting varied human-made environs, arthropod vectors are powerful engines of disease transmission [2]. Vector life history and ecological traits affect vector interactions with the vertebrate host as well as exposure to pathogens. However, pathogen development and/or amplification necessary for successful transmission into a new host, depend on vector physiology. Vector susceptibility to human pathogens, such as that of anopheline mosquitoes to human *Plasmodium* spp. is treated primarily as a vector trait, possibly because it is measured by the proportion of vectors that successfully carry developing parasites (prevalence) or the average number of parasites per vector (parasite load) following exposure to an infectious host. Vector susceptibility is commonly used interchangeably with the term “vector competence”; in relation to *Plasmodium* parasites, they refer to the ability of a mosquito species (or population) to support the development of *Plasmodium* parasites from gamete fertilization in the midgut through invasion of ookinetes, development of oocysts, and accumulation of infectious sporozoites in the salivary glands. Extensive literature describes variation in vector susceptibility to different parasite species [3], [4], [5], [6], [7], [8], [9].

The view that intrinsic vector factors are ultimately the most important in determining vector susceptibility is reflected in the medical entomologists' dream of controlling vector-borne diseases through the genetic manipulation of vector populations to render them resistant to the parasite they transmit [10], [11], [12], [13], [14]. However, vector susceptibility clearly is a composite trait expressing the joint occurrence of the two species with genetic contributions of both the parasite and the vector, as well as an environmental contribution (including environment-genetic interactions). Surprisingly, while several studies suggest that that compatibility varies between genotypes of parasites and vectors [15], [16], [17], we cannot find a single study that attempted to quantify the specific contributions of the parasite, the vector, and the environment to “vector susceptibility”.

Vector susceptibility is typically measured by the fraction of vectors becoming infectious following exposure i.e., those that support parasite development through to the stage capable for transmission to a new vertebrate host. Being a composite trait, it reflects both (i) purely vector determinants, termed: “narrow sense vector susceptibility” (nVS), (ii) purely parasite determinants, termed, “parasite compatibility” (nPC), as well as environmental effects. Here we define “broad sense vector susceptibility” (bVS), as the combined effect of nVS and nPC. Using separate selection experiments, we estimated the genetic contributions of *Anopheles stephensi* susceptibility to *Plasmodium gallinaceum* (nVS) and the parasite compatibility (nPC) with that vector, whilst attributing any unaccounted variation to environmental factors. We conducted separate selection experiments on mosquito susceptibility and on parasite compatibility (by selecting on the mosquito and the parasite, respectively) which allowed us to estimate the realized heritability (h^2^_r_) of each “side” while holding the other unchanged. The genetic contributions of the mosquito and the parasite were then compared to each other to assess if bVS in our system was primarily a vector trait (nVS), a parasite trait (nPC), or a consequence of certain environmental effects (including uncontrolled environmental variation). This approach provides a useful framework to deal with “vector” susceptibility comprehensively; otherwise major components of this important phenomenon are ignored and ineffective disease-control strategies may be devised. For example, the investment in genetically-modified mosquitoes rendered resistant to malaria or to dengue may fail if the evolution of parasite compatibility with those genotypes or with currently secondary vectors is ignored.

As with all estimates of heritability [18], those of nVS and nPC are relevant only to the particular system under study. Thus, other isolates of *P. gallinaceum* and/or other colonies of *An. stephensi* (or their respective natural populations) may provide different estimates if subjected to the same experiments. However, independent studies on one organism have revealed consistent results in most cases [18]. For example, heritability of abdominal bristle number in *Drosophila melanogaster* varied between 0.48 and 0.53 when estimated using offspring-parent regression, full-sib, and half-sib correlation methods, and realized heritability measured over ten generations of selection for increased weight in six-week old mice varied between 0.25 and 0.46 among six independent lines [18].

The avian malaria parasite *P. gallinaceum* was chosen for these experiments because it is a well-characterized laboratory system and unlike a rodent host, allows feeding of hundreds of mosquitoes on a single infected host as required in selection experiments which target a small fraction of the population. This parasite source might underestimate the “general” relative contribution of parasites to vector susceptibility (bVS) because this isolate might lack genetic variation (see below), but it could provide a minimal estimate of the parasite unique contribution to this trait. Moreover, our experimental design might be revealing if applied to more natural situations. The native vector for *P. gallinaceum* is believed to be a species of *Mansonia* [19], which is difficult to raise in the laboratory and the natural host is the junglefowl, presumably *Gallus lafayetii* [20]. In the laboratory, the common vector is *Aedes aegypti* and the common host is the domestic chicken (*Gallus gallus*). Upon infection with *P. gallinaceum*, *Ae. aegypti* prevalence is typically above 90%, whereas that of *Anopheles* mosquitoes is below 10% [21], [22]. For our experiment, we used the NIH colony of *Anopheles stephensi*, which routinely exhibits 0–5% prevalence after feeding on the same infected chicken that produces >85% infection in *Ae. aegypti*. Although not a natural vector-parasite system, the persistently low infection prevalence of independent *An. stephensi* colonies [21], [22], [23] indicated the presence of standing genetic variation in the parasite, the vector or both. Finally, such an artificial system represents a novel encounter between vector and parasite as typically accompanies a range expansion of either the vector or parasite.

## Materials and Methods

To select lines of *An. stephensi* susceptible to *P. gallinaceum* infection, we set up a traditional truncation selection, allowing only infected mosquitoes to breed whilst keeping the parasite strain constant in every generation (detailed below). In contrast, selection on *P. gallinaceum* for compatibility in *An. stephensi* was undertaken by passing infection from chicken to chicken using *An. stephensi* instead of *Ae. aegypti*. All mosquitoes used in this experiment were derived from a large breeder colony (1000–2000 parents per generation), that were never exposed to *P. gallinaceum*. Presumably this passage exerts selection on standing genetic variation influencing parasite compatibility with that vector since only compatible parasites to this vector can be passed to the next vertebrate host; thus we expect an increase in the frequency of infected *An. stephensi* mosquitoes over generations due to increased parasite compatibility alone (the mosquitoes were free of selection in this experiment). We use the term “generation” to denote infection cycle (or passage) whereby truncation selection was performed, even though the parasite underwent several multiplications in the chicken or in the mosquito from inoculation and until the selection took place.

To accommodate variation in mosquito infection parameters due to chicken infectivity, *Ae. aegypti* were fed on every infectious chicken. This allowed us to utilize the actual and the median of *Ae. aegypti* prevalence (95%) to standardize the prevalence in *An. stephensi*. Likewise, the actual and the median oocyst load was adjusted based on the corresponding median in *Ae. aegypti* (19 oocysts/gut; SD = 9.51).

### Mosquito maintenance and selection of *P. gallinaceum* susceptible lines

**Figure 1:**
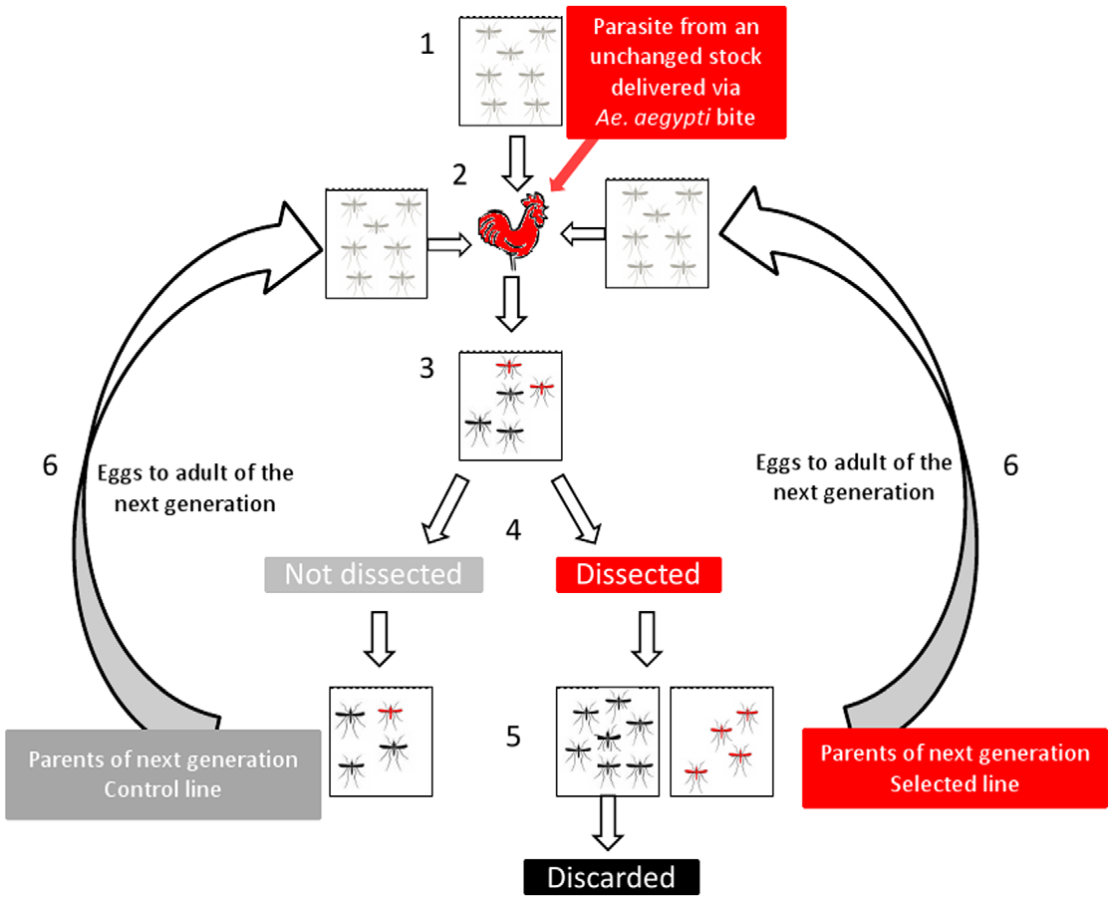
Selection protocol for increasing vector susceptibility. Schematic illustrating selection protocol for increased vector susceptibility (nVC) in *An. stephensi* infected with *P. gallinaceum*. (1) *An. stephensi* colony mosquitoes randomly chosen for the selection experiment; (2) feed on *P. gallinaceum* infected chicken (side by side with *Ae. aegypti*, used as positive control); (3) Mosquitoes separated out individually on day 5 p.i for oviposition. On day 6 p.i., a subset of the females that laid eggs (50<N<200) were dissected for determination of oocyst count in their midgut i.,e., red denotes infected and black denotes uninfected. (4) Eggs set up from all infected mosquitoes (red) to generate the next generation of the selected line and from a matching number of unknown (not dissected) females to generate the next generation of the control line (gray). (5) Larvae reared to adults for next cycle. (6) Offspring of the selected and control line fed again on an infected chicken. Processes 2–6 repeated for subsequent generations.

Mosquitoes were reared under 28°C, 75% humidity, and 12 hour light/dark cycle. For the selection experiment, 3–8 days old female mosquitoes were first separated out into groups of 300–400 individuals per cage. Experiments comprised of mosquitoes which were 1–3 days apart in age. Mosquitoes were maintained on distilled water for 12–15 hours prior to feeding on a restrained infected chicken (parasitemias normally between 10–20% with gametocytes present) for up to 45 minutes depending on feeding rate (90% of females typically feed within 20 minutes). A few hours post-feeding, unfed females were removed. A schematic describing the selection process is shown in Figure 1.

Following feeding, mosquitoes were given sugar solution daily and maintained under standard insectary conditions. On day four post-infection (p.i.) they were separated out individually into labeled 50 ml tubes lined with absorbent paper and topped with netting. On day 5 p.i., 10 ml of water was added to each tube and egg lay was monitored over two days (days 6 and 7 p.i.). Infection status was determined only in mosquitoes that laid eggs by dissecting their midguts and counting all oocysts (200× magnification). Care was taken during dissection to ensure the identity of each female was known and that no egg transfer occurred during removal from the 50 ml tube.

Eggs from all dissected mosquitoes that were positive for *P. gallinaceum* infection were pooled and reared under standard insectary conditions (Lines A and E). A matching number of undissected females were randomly chosen to initiate control lines (Lines C and F) and their eggs were set up following the exact conditions of the offspring from infected mosquitoes.

Adults of the F_1_ generation of the selected and control lines were given sugar solution daily and offered an uninfected bloodmeal three and four days after the adult emergence. Egg dishes were placed in the cages three days later. Blood feeding of F1 adults was repeated for amplification of the F1 generation so sufficient offspring were available for a second round of selection. The second round of selection was carried out following the same procedure as the first round.

Subsequent generations of each line were reared as for F_1_, but amplification (i.e., feeding on uninfected chicken to increase the number of adult offspring needed for selection experiments) was only used when numbers were low. In lines A and C, suitable numbers of offspring were available from the third selection generation onwards and no amplification between selection experiments was required. In lines E and F, amplification between selection experiments was required until generation six. To ensure similar effective population size for the control and selected lines, matching numbers of bloodfed females in experimental and control lines were used. Typically the eggs from 70–100 females were reared in each generation. Selection was stopped once prevalence exceeded 85%, at which time mosquitoes were maintained under relaxed selection as required for additional experiments that will be published separately.

### Parasite maintenance and selection for compatibility with *An. stephensi*

**Figure 2:**
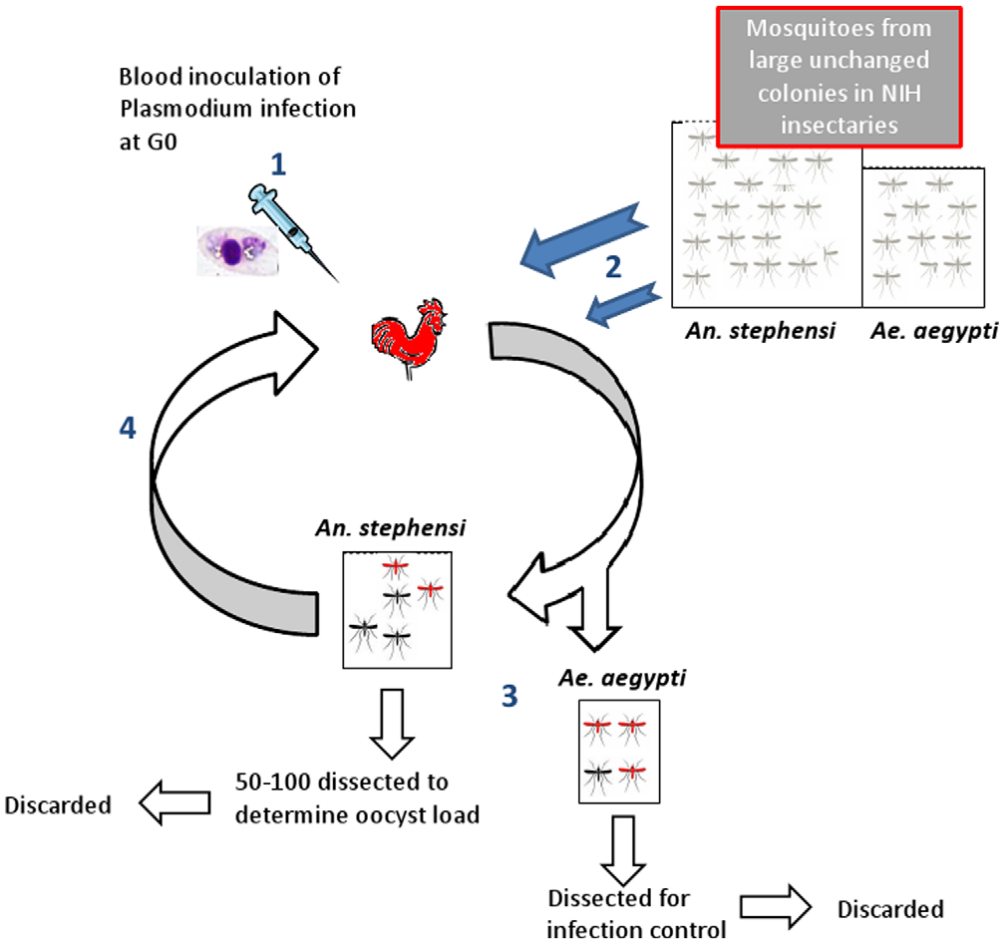
Selection protocol for increasing parasite compatibility. Schematic illustrating selection protocol for increased parasite compatibility (nPC) of *P. gallinaceum* infecting *An. stephensi*. (1) *P. gallinaceum* stock was injected into a chicken host, which was monitored until parasitemia reached near 10–20%. (2) Approximately 700 female *An. stephensi* mosquitoes randomly chosen from a large stock colony were fed on the infected chicken (side by side with 30–50 *Ae. aegypti*, used as positive control). (3) On day 6 p.i., a subset of the *An. stephensi* females (50<N<100) were dissected for determination of oocyst load in their midgut i.,e., red denotes infected and black denotes uninfected. (4) Infection of a new chicken by the parasite that completed its development in *An. stephensi* was produced by feeding of the remaining ∼500 females on a new chicken host. The next parasite selection cycle started from step 2. Note that parasite selection is totally independent from mosquito selection because no infected mosquitoes were allowed to produce offspring.

*P. gallinaceum* (8A strain) was routinely maintained by continuous cycles in white leghorn chickens (*Gallus gallus*) and frequent passage by *Ae. aegypti*. Isolated from one jungle fowl around 1936 [20] it has passed hundreds of times between chickens. Therefore, this parasite source might have lost much of its genetic diversity during its adaptation to the domestic chicken and to *Ae. aegypti*, as well as during the multiple passages it underwent since isolation (however, no independent source of this parasite is known to us). To select a parasite line compatible with a new mosquito vector, parasite passages between chicken hosts were carried out exclusively by *An. stephensi* (Figure 2). To maximize the chances of successfully transmitting the infection from *An. stephensi* to naïve chickens, large numbers of mosquitoes per cycle (>600) were used. Females were separated into two cages each containing 300–350 mosquitoes and were fed simultaneously for up to 45 minutes on the infected chicken. A smaller cage of *Ae. aegypti* was fed immediately afterwards in order to standardize prevalence in *An. stephensi* (see below). Following feeding, unfed *An. stephensi* were removed and mosquitoes were maintained under standard insectary conditions. Egg bowls were provided on days 4–5 p.i., but all eggs were discarded.

Infection was monitored on day seven p.i. by dissecting 70–100 mosquitoes. The remaining mosquitoes were maintained for infection of a new naïve chicken on day 12. The mosquitoes were fed again on the same chicken on day 14 p.i to maximize the chance of passing infection onto that naïve host. *Ae. aegypti* were fed only once on each chicken as described before. Infected chickens were tagged upon exposure and parasitemia was monitored by daily bloodsmears from day seven onwards. Infection of naïve mosquitoes was carried out when the total parasitemia in the chicken was 10–20% and gametocytes were observed. Following feeding, chickens were exsanguinated via heart puncture and parasites were cryopreserved following standard protocols [24]. Because of the long generation time and high workload, only a single selected line of the parasite was produced.

### Statistical Analysis

Realized heritability (h^2^_r_) is a measure of the additive genetic contribution to the phenotypic variation in a given trait. As is typical for quantitative traits, h^2^_r_ of oocyst load (range: 0 to 250 per mosquito) was estimated by regressing the cumulative response to selection on the cumulative selection differential [18] as briefly explained below. The response to selection (R) was estimated in each line as the difference in mean phenotypic value between that of the offspring and parental population. The selection differential (S) was estimated as the difference in mean phenotypic value between that of the selected parents (subset) and their respective total population from that generation. Cumulative values of R and S were calculated as the sum over all previous and current generations (of selection), respectively. Because selection on the vector was performed on single parents (mothers), the slope coefficient measuring the change in offspring oocyst load (R) in relation to the change in their parents' oocyst load by the selection (S) was doubled (vector only), assuming equal contribution by both parents, following R = h^2^_r_ S [18].To monitor and accommodate variation in infection of *An. stephensi* due to chicken parasitemia, *Ae. aegypti* were used as a positive control. Selection on vector susceptibility was performed when prevalence of *Ae. aegypti* was greater than 80%, except in the 10^th^ and 11^th^ generations of the selection on parasite compatibility with prevalence of 78 and 79%. Further, infection measures in *An. stephensi* were standardized by the ratio of the actual to the median prevalence and oocyst load in *Ae. aegypti*, respectively.

**Figure 3:**
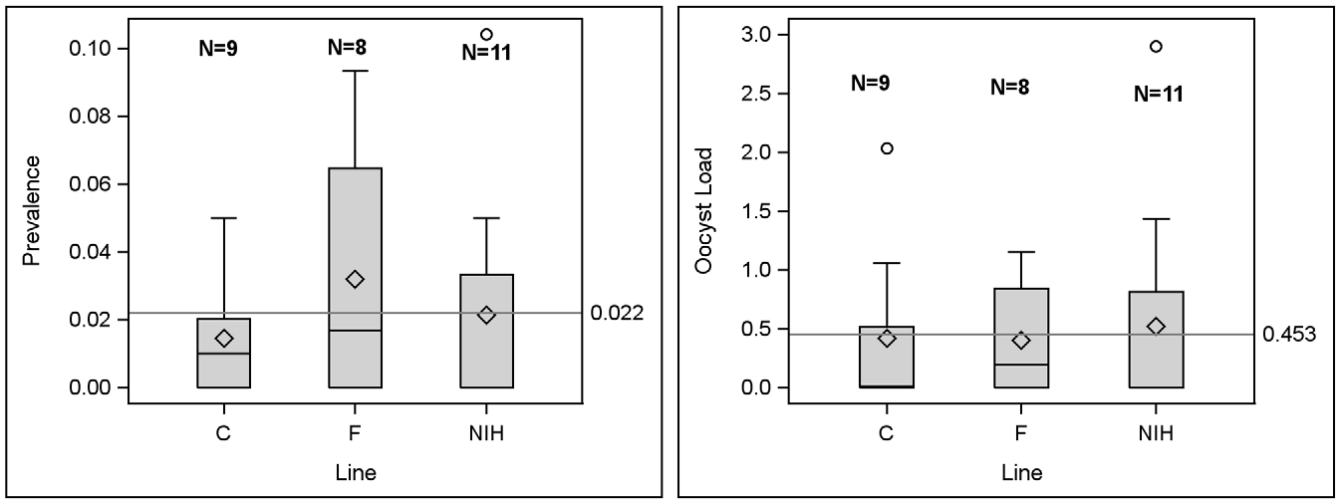
Infection prevalence and intensity in unselected lines of *An. Stephensi*. *Anopheles stephensi* prevalence and mean oocyst load in unselected lines over a total of 28 infection experiments. Overall mean is shown by the horizontal line. Number of experiments is shown above each box-whisker plot (sample size range per experiment 50–175, except n = 20 in one experiment with the NIH line). The differences among lines in overall prevalence was not significant (χ2 = 3.96, df = 2, P<0.137) as was the case for the oocyst load (ANOVA with Experiment treated as blocking factor: F_2,2135_ = 0.58, P>0.55).

**Figure 4:**
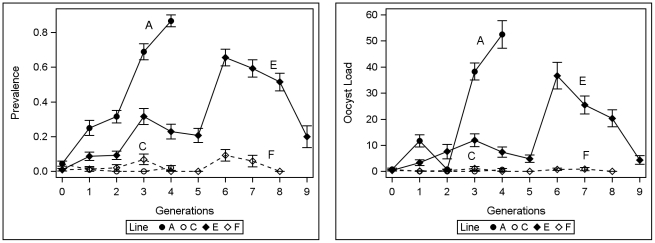
Infection prevalence and intensity in selected lines. The changes in the prevalence and mean oocyst load in the selected lines (A and E) of *An. stephensi* and their corresponding controls (C and F) over generations. Vertical bars represent standard error of the means (SEM, calculated separately for each generation). Only four generations were required by the A line to reach its target phenotype (prevalence>85%), whereas the E line did not reach this target after 9 generations, when the experiment was terminated.

As part of the selection on the vector, an *An. stephensi* control line was maintained side by side with each selected line, keeping the number of mothers for each generation of selection the same to assess the effect of random drift and systematic environmental change. We did not subtract the control line from the selected line because the prevalence of control line remained stable 0–5% (Figures 3 and 4).

Unlike parasite load (above), host infection status is a discrete dichotomous trait (i.e., a state of infected vs. uninfected), which is typically analyzed using the liability model [18]. This model assumes that the state is determined by the underlying liability trait, which is shaped by multigene effects. If the individual value of the liability exceeds a threshold value (T) the phenotypic state will change. Liability is assumed to be normally distributed, hence the mean liability of a population (mu) can be estimated from the fraction exhibiting infection (q = prevalence or the fraction of mosquitoes with 1 or more oocysts). The probit transformation of q was computed in SAS [25]. Selection differential was calculated as S = ((mu^SelParent^−mu^TotlParent^)/q))*((p−q)/(1−q)) where p is the fraction of individuals exhibiting the state (infected) among the selected parents (equation 10.32 [26]). Because only infected females were included in the selected parents, whilst the male's phenotype could not be determined, the total parental liability was estimated assuming that every generation male liability was equal to that of the females and that mating was random. Accordingly, p was calculated as p = (1+q)/2.

### Ethics Statement

This study was carried out in strict accordance with the recommendations in the Guide for the Care and Use of Laboratory Animals of the National Institutes of Health. All procedures were approved by the National Institutes of Health Animal- Care and Use Committee (ACUC, Protocol ID: LMVR102). 

## Results

### Selection on susceptibility of *An. stephensi* to *P. gallinaceum*

Before selection, prevalence of *An. stephensi* after feeding on chickens infected with *P. gallinaceum* was 2% (Number of experiments = 28, range: 0–10%, Figure 3) and the mean oocyst load was 0.45 (range: 0–3). Each of the two selected lines (A and E), had its respective control line (C and F), which was treated identically and maintained in the same insectary (side by side). Because fluctuations in the prevalence and oocyst load of the control lines were minimal and no systematic change was detected between them and the large (unselected) NIH colony (Figure 3, P>0.13, and below), analyses were performed directly on the selected lines rather than on the difference between selected and control line. In contrast to the control lines (C and F), the selected lines (A and E) exhibited a systematic change in susceptibility (Figure 4). Only four generations were required by the A line to reach its target phenotype (prevalence>85%), whereas the E line responded to selection, but did not reach this target after 9 generations, after which the experiment was terminated.

**Figure 5:**
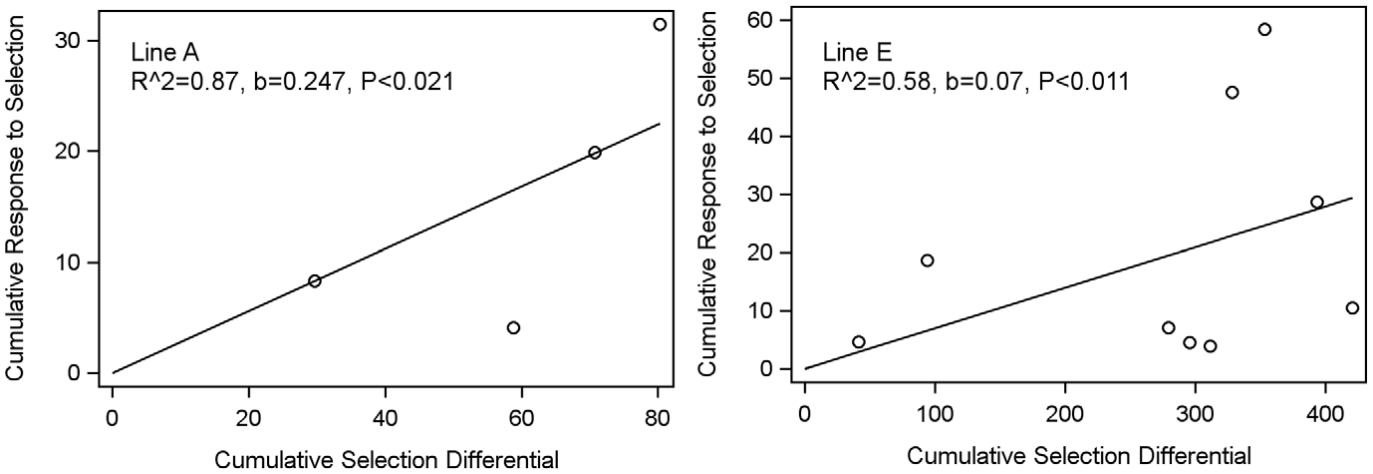
Response of *An. stephensi* selected for susceptibility to *P. gallinaceum* based on mean oocyst load. The cumulative response to selection is regressed on the cumulative selection differential for the selected *An. stephensi* lines. Regression is forced through the origin.

Vector infection prevalence was the primary focus of our experiment, as truncation selection was applied against all mosquitoes with zero oocysts (see Materials and Methods). The estimated realized heritability of *An. stephensi* to its “vector susceptibility” (nVS), measured by infection prevalence, was high (mean value of 0.66, Table 1). As can be expected from the phenotypic response to selection (Figure 4), the realized heritability of the A line (0.83) was higher than that of the E line (0.49). Likewise, for oocyst load, h^2^_r_ of line A (0.48, twice the slope coefficient [b] in Figure 5) was higher than that of line E (0.14, Figure 5) consistent with their differing responses to selection as illustrated in Figure 4. The average genetic contribution of the vector (nVS) to “vector susceptibility” (bVS) as measured by oocyst load was 0.31 (Table 1).

**Table 1:** Effect of selection on mean oocyst load and prevalence of *An. stephensi* and *P. gallinaceum* and their realized heritability (h^2^_r_).

		Oocyst Load (adjusted)		Prevalence (adjusted)	
	t^a^	Z_0_/Z_t_ b	h^2^_r_	Z_0_/Z_t_ b	h^2^_r_
*An. stephensi* (Line A)	4	1.4/26.8	0.49	2.3%/80%	0.83
*An. stephensi* (Line E)	9	0.4/11.0	0.14	2.3%/74%	0.49
*An. stephensi* (Overall)		—	0.32		**0.66**
P. gallinaceum (Line 1)	12	0.01/1.8	0.02	1%/5%	**0.05**

a Denotes the number of generations selection was carried out.

b Phenotypic values before selection (**Z_0_**) and after the last generation of selection (**Z_t_**).

### Parasite Selection

**Figure 6:**
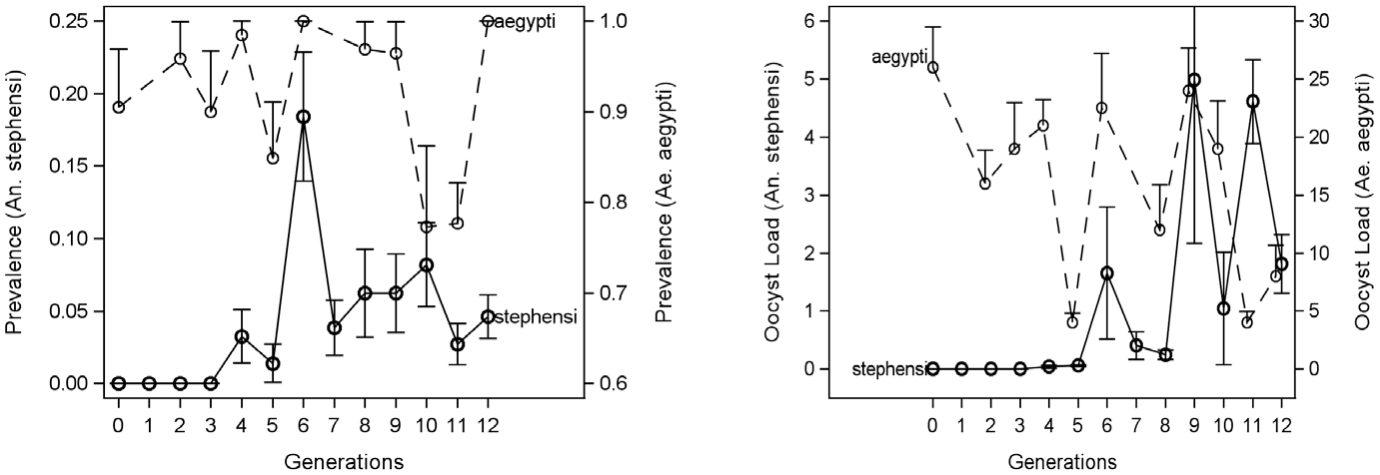
The changes in prevalence and intensity of infection over parasite selection. The changes in the prevalence and mean oocyst load following selection in a line of *P. gallinaceum* vectored exclusively by *An. stephensi* compared with that in *Ae. aegypti* that was simultaneously fed on the same chicken. Note the different Y-axes for the different mosquito species. Prevalence values of *An. stephensi* were adjusted by the mean prevalence of *Ae. aegypti* (0.95). Values of oocyst load of *An. stephensi* were adjusted by the mean oocyst load of *Ae. aegypti* (19.5). Vertical bars represent SEM (calculated separately for each generation). To avoid clutter, bars representing mean-SEM of *Ae. aegypti* were excluded.

Selection on the parasite for compatibility with *An. stephensi* (nPC) was undertaken by continual passage of infection from chicken to chicken by *An. stephensi* instead of the typical laboratory vector *Ae. aegypti*. Because we used mosquitoes from a large breeding colony that were not subjected to selection for parasite susceptibility, only the parasite could respond to its “new” vector i.e., there was no systematic reproductive differential between “selected” and “not selected” vectors. The response of the parasite to this selection was measured by the change in prevalence and in parasite load in the new vector. The parasite's response was more erratic than that exhibited by the mosquito lines under selection (Figure 6). In particular, it is difficult to explain the remarkably high prevalence in the 6^th^ generation and the high oocyst loads in the 9^th^ and 11^th^ generations.

**Figure 7:**
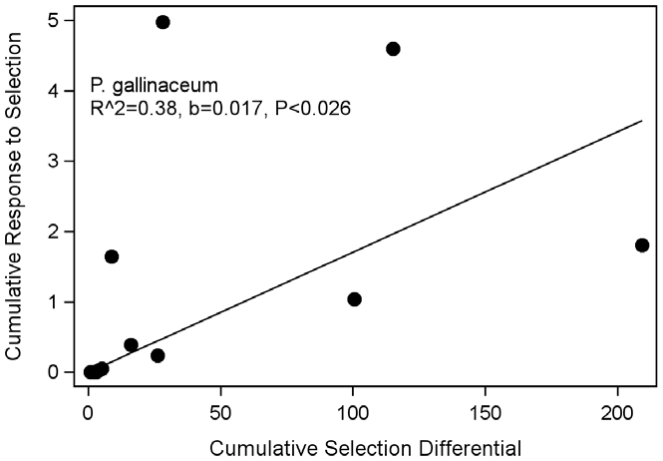
Response of *P. gallinaceum* selected for compatibility with *An. stephensi* based on mean oocyst load. The cumulative response to selection is regressed on the cumulative selection differential for the selected *An. stephensi* lines. Regression is forced through the origin.

Realized heritability of parasite compatibility (nPC) based on the standardized oocyst load was calculated as described above (see Materials and Methods). The heritability estimate of oocyst load was rather low (2%, see the slope coefficient in Figure 7). The heritability of the prevalence was not considerably higher (5%, Table 1), indicating that the genetic contribution of *P. gallinaceum* to the variation in susceptibility of *An. stephensi* (bVC) was rather small.

## Discussion

Variation in vector susceptibility to parasites has been a central concept since Huff's selection experiments on resistance of *Culex pipiens* infected with bird malaria [27]. The production of resistant lines in that and other vector species against certain parasite isolates has lead to the commonly-held view that “vector susceptibility” is ultimately a trait of the vector. Evaluating whether the resistance held true against other parasite isolates has never been done to our knowledge. That the parasite was not allowed to evolve in these experiments was deemed irrelevant. Moreover, no studies have demonstrated that parasite selection for compatibility with a particular vector is bound to fail, i.e., could not yield highly compatible parasites against lines that were considered non- or poor vectors. On the contrary, a rapid adaptation of *Brugia patei*, a filarial nematode, to a new mosquito vector, *Ae. togoi*, was reported whereby the prevalence of the vector increased from 40% to 90% in four generations of selection [28]. There is ample evidence of parasite species switching hosts most notably in malaria parasites of avian and primate hosts e.g., [29], [30], [31], [32], [33], [34] and indeed the spread of malaria parasites to new geographic regions must have occurred by the rapid adaptation of the parasites to new vectors (e.g., [29]). Thus, a practical approach to consider the specific contribution of the parasite as well as the vector is needed.

The current study is, to our knowledge, the first in to assess the specific contribution of the parasite (nPC), the vector (nVS), and also infer the contribution of the environmental variation (broadly defined to include genetic-environment interactions) on the variation in vector susceptibility (bVS). In this study, short-term selection experiments, performed independently on the vector and the parasite, were used to assess the respective (realized) heritabilities of the (i) prevalence and the (ii) parasite load of *An. stephensi* infected with *P. gallinaceum*. Parasite load in infected mosquitoes would be a better measure than parasite load in all mosquitoes because it decouples the inherent correlation between the prevalence and overall parasite load. It would be especially valuable in macroparasites such as filariae, which undergo development in the vector without amplification. Our experimental design, however, did not allow us to estimate the heritability of the oocyst load in infected mosquitoes because we allowed breeding of only positive females. Hence, we could not estimate the selection differential in this trait.

Our results indicate that, in this system, the largest component of vector susceptibility (bVS) was indeed the vector's (67%), vs. the 5% parasite-specific contribution. The environmental component was deduced by subtracting the former from 100% assuming non-additive genetic component to be negligible. It appears contradictory that the estimate of the environmental effect was near 30% when inferred from the selection on the vector, yet it was near 95% when inferred from the selection on the parasite. However, the environmental effect is the unaccounted variance after accounting for the genetic component reflecting the response to selection, i.e., heritability. Thus it is not surprising that the estimates of the environmental effect are so different. Further, this environmental component includes genetic-by-environment interactions (GxE) in addition to “effects of uncontrolled environmental variation” [35], [36], [37]. Surprisingly, the total contribution of the environment was approximately 30%, which is considerably greater than expected. As in most laboratory studies, environmental variation was minimized compared with natural environmental variation; thus, this is a conservative lower-limit estimate for the environmental variation under field conditions. An important implication of a large contribution of the environment is that genetically “resistant” genotypes of the vector in the laboratory might, under certain environmental conditions, act as good vectors in the field. More work must be devoted to understand this possibility and if it is subject to certain limits. Importantly, the large contribution by the vector's genetics (as opposed to the parasite's) is consistent with the conventional notion that vector susceptibility is primarily a vector trait, but additional explanations which are not consistent with this notion cannot be ruled out, as discussed below.

Because our *P. gallinaceum* originated from one jungle fowl isolated over 70 years ago [20], was adapted to the domestic chicken and to *Ae. aegypti*, and was subjected to multiple bottlenecks throughout its history, it likely resembles a single clone today. Accordingly, the absence of standing genetic diversity in the parasite would limit its response to selection and underestimate the parasite's potential response compared to a natural population with more typical genetic diversity [38]. If this explanation is true, different parasite genotypes would possess different compatibility with mosquito genotypes [15], [39]. Consequently the response to selection by isolates containing diverse genotypes would be greater than observed here. We could not locate a single independent isolate of *P. gallinaceum* to test this hypothesis. If new favorable mutations with respect to our selection pressure were generated de novo during the experiment, they were not picked up by our mosquitoes, suggesting they were too rare.

For the parasite, compatibility with the vector is a primary fitness trait much like egg production for a female mosquito and sperm production for the male. This results in the most intense natural selection [38], [40], [41], which typically depletes standing genetic variation in genes underlying it [42]. On the other hand, resistance is of secondary importance to vector fitness because exposure to *Plasmodium* is usually low (<10%, [43], [44], [45], [46], [47], [48], a factor compounded by the modest impact of infection on vector survival and reproduction (<30%; [49], [50]). Unlike typical insect pathogens, vector-borne parasites must limit their impact on the vector since their transmission is linked with vector survival, motility, and biting behavior [51], [52]. If true, short-term selection studies “inflate” the role of the vector, even though the evolution of susceptibility is primarily mediated by the parasite. The consequence of this scenario is that parasite evolution would quickly overcome resistant genes in the vectors or potentially “spill over” to other mosquito vectors if their primary vector became resistant.

Our current study illustrates the problem inherent in the term vector susceptibility if it is treated as a vector trait, and outlines an approach for its resolution. Although this study provides information on a specific system of an unnatural vector-parasite combination, its main merit is the development and demonstration of an experimental design that could be applied to other parasite-vector and parasite-host systems. Future studies should use genetically-diverse vector and parasite sources for experiments and maximize replicate lines as logistically possible. Only the accumulation of additional studies on natural and unnatural vector-parasite systems will be helpful in determining which of these three explanations holds true to vector-parasite systems or to particular systems of interest, e.g., *P. falciparum* and *An. gambiae*. Resolving between these explanations namely that (1) vector susceptibility is ultimately a vector trait, (2) co-adapted parasite genotypes co-occur with those of the vector, and (3) that in the longer evolutionary context, vector susceptibility is primarily a parasite trait, have important implications for disease control. If indeed one of the last two explanations holds true to the particular system under consideration, genetic control efforts will probably be ineffective, because a resistant vector is expected to be susceptible to certain parasite genotype(s) or is expected to be met by a new mutant(s) which, due to the intense selection will spread rather quickly once a “resistant vector” is introduced. The methodology outlined here can be helpful in resolving these options.
